# Human Induced Pluripotent Stem Cell-Derived Vascular Cells: Recent Progress and Future Directions

**DOI:** 10.3390/jcdd8110148

**Published:** 2021-11-04

**Authors:** Jee Eun Oh, Cholomi Jung, Young-sup Yoon

**Affiliations:** 1 Severance Biomedical Science Institute, Yonsei University College of Medicine, Seoul 03722, Korea; jeeeunoh@yuhs.ac (J.E.O.); cjung90@yuhs.ac (C.J.); 2 Research and Development Center, KarisBio Inc., 50-1 Yonsei-ro, Avison Biomedical Research Center Room 525, Seodaemun-gu, Seoul 03722, Korea; 3 Department of Internal Medicine, Graduate School of Medical Science, Brain Korea 21 Project, Yonsei University College of Medicine, Seoul 03722, Korea; 4 Department of Medicine, Division of Cardiology, Emory University School of Medicine, Atlanta, GA 30322, USA

**Keywords:** human induced pluripotent stem cell, endothelial cell, smooth muscle cell, stem cell, cardiovascular disease, regenerative medicine

## Abstract

Human induced pluripotent stem cells (hiPSCs) hold great promise for cardiovascular regeneration following ischemic injury. Considerable effort has been made toward the development and optimization of methods to differentiate hiPSCs into vascular cells, such as endothelial and smooth muscle cells (ECs and SMCs). In particular, hiPSC-derived ECs have shown robust potential for promoting neovascularization in animal models of cardiovascular diseases, potentially achieving significant and sustained therapeutic benefits. However, the use of hiPSC-derived SMCs that possess high therapeutic relevance is a relatively new area of investigation, still in the earlier investigational stages. In this review, we first discuss different methodologies to derive vascular cells from hiPSCs with a particular emphasis on the role of key developmental signals. Furthermore, we propose a standardized framework for assessing and defining the EC and SMC identity that might be suitable for inducing tissue repair and regeneration. We then highlight the regenerative effects of hiPSC-derived vascular cells on animal models of myocardial infarction and hindlimb ischemia. Finally, we address several obstacles that need to be overcome to fully implement the use of hiPSC-derived vascular cells for clinical application.

## 1. Introduction

Ischemic cardiovascular disease is the leading cause of global morbidity and mortality [1,2,3]. In a significant population of patients, existing surgical and interventional treatments in combination with medical therapy are not effective because the anatomical complexity, aging, multiple procedures, and comorbidities may leave patients with no option, or at least poor options, for revascularization [4,5,6,7,8]. Such clinical limitations justify the search for an alternative therapeutic option that may beneficially influence the outcomes of these no-option patients.

Cell-based therapy offers an unprecedented opportunity to treat ischemic cardiovascular disease that requires revascularization. Its mechanism is mainly governed by the concept of neovascularization, which means formation of new blood vessels [9,10]. Neovascularization occurs through three distinct processes: vasculogenesis, angiogenesis, and arteriogenesis (reviewed in detail by Carmeliet [11], Jain [12], and Simons [13]). Briefly, vasculogenesis refers to the de novo formation of the primitive vascular plexus from stem or progenitor cells during embryogenesis [14], whereas angiogenesis is a process through which new capillaries are formed not only from pre-existing vessels but also by proliferation, sprouting, and migration of endothelial cells (ECs) [15,16]. Lastly, arteriogenesis is the extensive growth of relatively larger vessels, such as arterioles and arteries [17,18]. In arteriogenesis, smooth muscle cells (SMCs) are recruited to cover the EC layer, thereby resulting in vessel maturation and stabilization [19,20,21,22]. While ECs are the most basic building block of blood vessels and capable of forming capillary-like vessels, SMCs are unequivocally necessary for the development of a mature and durable vasculature [11,13,23,24,25]. Thus, cell-based therapy using vascular cells, such as ECs and SMCs, is an approach to induce therapeutic neovascularization, which not only rebuilds a vasculature but also restores blood perfusion and cardiac or vascular function.

Earlier studies used a myriad of adult stem or progenitor cells to induce neovascularization. While effective in animal models of myocardial infarction [26,27,28,29] and hindlimb ischemia [30,31,32], clinical trials have produced controversial results [33,34,35,36], with modest effects at best. Mechanistically, adult stem or progenitor cells exert therapeutic effects, mainly through paracrine effects [37,38,39]. Accordingly, the use of human embryonic stem cells (hESCs) was proposed as a new therapeutic option for cardiovascular regeneration. Human ESCs, which are the inner cell mass of the blastocyst, are pluripotent and can differentiate into any cell type of the ectodermal, mesodermal, and endodermal lineage [40,41]. Furthermore, hESCs can maintain pluripotency and proliferative capacity [42]. Differentiation of hESCs into ECs and SMCs have been demonstrated [43,44,45,46,47]. While a plethora of studies have revealed the experimental promise of hESC-derived vascular cells in animal models of myocardial infarction [48,49] and hindlimb ischemia [50,51,52], their clinical application is significantly hampered by immunogenicity due to allogeneic use and ethical concerns about obtaining human embryos.

Yamanaka and colleagues reported generation of human induced pluripotent stem cells (hiPSCs) in 2006 [53]. This new cell type has opened a new era of stem cell biology, cell-based therapy, and regenerative medicine. Originally, human iPSCs (hiPSCs) were generated from adult human dermal fibroblasts with retroviral vectors containing four transcription factors, POU5F1 (OCT4), SOX2, KLF4, and MYC [54]. Thereafter, various cell sources such blood [55,56,57,58] and urine [59,60], other inducing factors such as LIN28, NANOG, and microRNAs [61,62] and other delivery vectors including plasmids [63,64] and modified mRNAs [65,66] have been developed, allowing clinical application [67]. Human iPSCs are similar to hESCs in molecular and genetic features, morphology, pluripotency and unlimited proliferation potential. Of note, they have additional advantages over hESCs in that they are free of ethical controversy and autologous cell generation is possible. With these characteristics, hiPSCs have emerged as strong candidates for cell-based therapy. Owing to their multilineage differentiation potential, they have shown promising results in their differentiation into ECs and SMCs, which are crucial sources for vascular regeneration.

**Figure 1:**
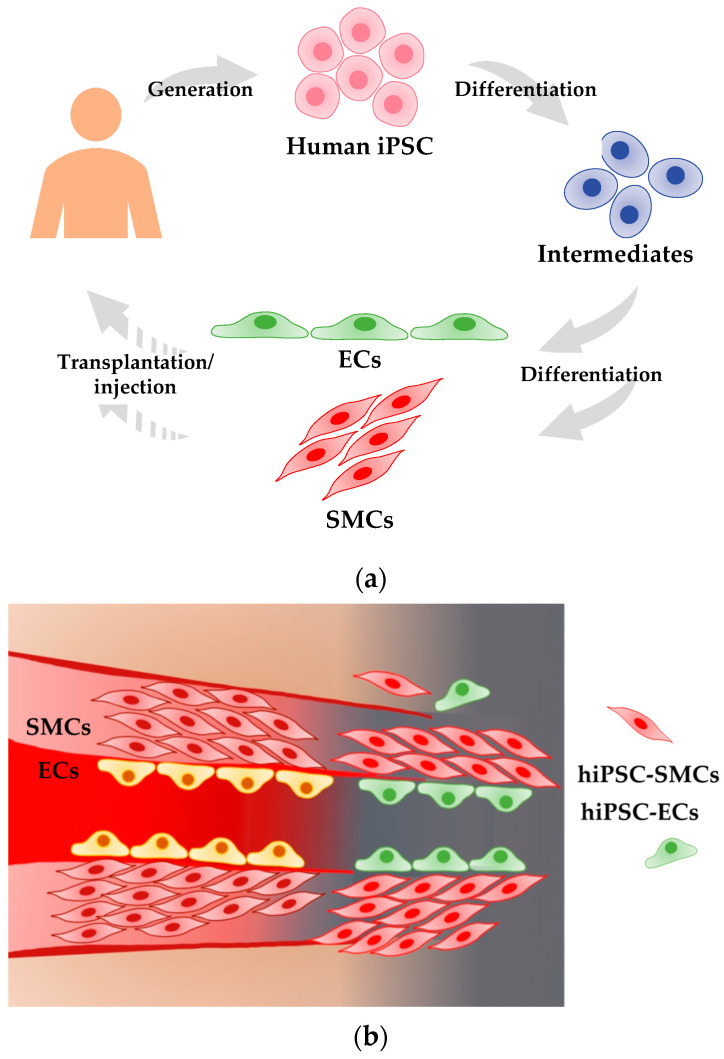
Proposed scheme for clinical application of hiPSC-derived vascular cells to treat patients with ischemic cardiovascular diseases. (**a**) Human iPSCs can be generated from somatic cells, such as skin fibroblasts and blood cells. Human iPSCs can differentiate into either ECs or SMCs via intermediates. The resulting vascular cells can be used for cell-based therapy to induce neovascularization. Solid arrows indicate verified and validated steps. Dashed arrows indicate speculative steps yet to be demonstrated. (**b**) Transplanted hiPSC-derived ECs incorporate into vessels, which are covered by hiPSC-derived SMCs, to form neovascularization in ischemic regions.

In this review, we discuss the methods for differentiating hiPSCs into ECs and SMCs, collectively called vascular cells. We then review criteria to define the identity of hiPSC-derived ECs and SMCs. We further explore the therapeutic potential of hiPSC-derived vascular cells in animal models of cardiovascular diseases. Lastly, we describe challenges for clinical application of these two types of cells (Figure 1).

## 2. Human Induced Pluripotent Stem Cell-Derived Endothelial Cells

In regenerative medicine, hiPSC technology has brought a new era for inducing neovascularization. Shortly after the first discovery of murine iPSCs and human iPSCs [53,54,68], studies showed successful differentiation of hiPSCs into ECs in 2009 [69]. Taura et al. evaluated the feasibility and compatibility of EC differentiation from four different hiPSC lines compared to three hESC lines. After this first successful EC generation, much effort was made to define and optimize hiPSC-EC differentiation processes. In this section, the advances in hiPSC-EC differentiation systems are reviewed, denoting EC features to postulate their functionality in neovascularization.

### 2.1. Differentiation of hiPSCs into ECs

**Figure 2:**
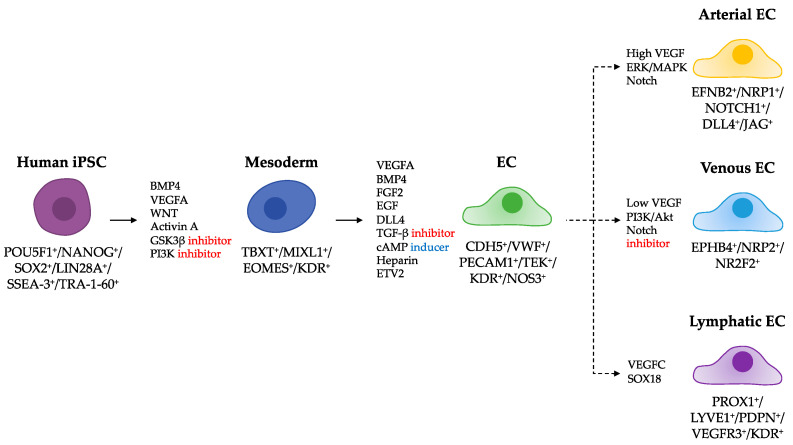
Proposed strategies to derive endothelial cells from hiPSCs via mesodermal lineages. Combinations of growth factors, cytokines, small molecules, transcription factors and phenotypic markers that define mesodermal cells or ECs are listed. Dashed lines indicate that hiPSC-derived ECs can be further differentiated into arterial, venous, and lymphatic EC subtypes.

Traditional differentiation methods of hiPSC-derived ECs include embryoid body (EB)-mediated and monolayer-directed approaches. EBs, aggregated masses of pluripotent stem cells in suspension culture, can spontaneously differentiate into ECs. However, due to the spontaneous process that leads three-germ layered EBs to differentiate into multiple lineage cells, the system holds two critical limitations: inconsistency and low yield. Although endothelial differentiation of hESCs through EB-mediated methods was first achieved as early as 2002, the differentiation yield was only 2% [44]. Likewise, the first hiPSC-derived ECs by Taura et al. yielded less than 5% of the total population [69]. In 2011, Rufaihah et al. [70] were able to differentiate hiPSCs into ECs with better efficiency, ranging from 5 to 20%, via the EB-mediated method. Supplemented with EC-inducing factors BMP4 and VEGF in the culture media, hiPSC-ECs that were positive for PECAM1, CDH5, NOS3, and VWF were generated. As the 3D protocols are laborious and complex, involving dissociation of 3D aggregates and further expansion in 2D monolayered culture [71,72], simple 2D differentiation systems without EB formation were developed. The 2D monolayered system often comprises coculture techniques with other cell types or feeder-free methods. However, because it is imperative to exclude contamination from feeder cells used for attachment, such as mouse embryonic fibroblasts (MEFs), we only discuss here the protocols using coating matrices such as collagen, Matrigel, or Fibronectin [73,74]. Moreover, the 2D differentiation system is advantageous over the 3D system because homogeneous exposure of culture media permits more consistent EC derivation and much improved yields [75]. Studies have shown that hiPSC-derived ECs using a simple 2D differentiation system resulted in efficiency exceeding 80–98% [73,76,77]. Here, we focus on EC-directed 2D differentiation approaches, which are organized into distinct stages via sequential treatments of defined conditions for growth factors, cytokines, and small molecules (Figure 2).

Adapted from mammalian embryo development, the first step for generating endothelial cells is to differentiate hPSCs into the mesodermal lineage [78,79,80]. In the embryo, the mesoderm layer gives rise to primordial vascular cells [14,81]. It was previously established that Wnt signaling is necessary for embryonic mesoderm induction, especially for early primitive streak formation and gastrulation, shown by studies of mice deficient in the Wnt3, Wnt coreceptors Lrp5 and Lrp6, and β-catenin [82,83,84]. Lindsley et al. then confirmed that canonical Wnt activity in vivo was conserved during the equivalent steps of ESC-derived mesoderm differentiation [85]. Upon inhibition of endogenous Wnt signaling, generation of Kdr^+^ mesodermal precursors and mature mesodermal lineages failed, and mesoderm-specific genes were not induced. In addition, a morphogen, BMP4, promotes gastrulation and ventral mesoderm formation and is responsible for the dorsal-ventral axis alignment [86,87]. It was also noted that BMP4, as a member of the TGF-β superfamily, initiates Wnt signaling pathways and activates activin/nodal pathways to generate the primitive streak in mice [88,89]. In hESC cultures, BMP4 also triggered Wnt and nodal signaling to generate early embryonic germ layers [90,91]. It was also reported that GSK3β inhibition by CHIR99021 or CP21R7 in combination with BMP4 treatment allowed rapid commitment to mesodermal cell fate [76]. Synergistic effects may be provoked because activation of canonical Wnt/β-catenin signaling initiates molecular cascades that ultimately inhibit GSK3β [92]. FGF2 is also used in mesodermal differentiation. In early *Xenopus* and zebrafish studies, it was demonstrated that FGF signaling also plays an important role in the formation and maintenance of axial and paraxial mesoderm [93,94,95,96]. Fletcher and Harland specified that FGF signaling did not affect axial mesoderm induction but maintenance, whereas it directly induced paraxial mesoderm formation [96]. PI3K catalytic activity, acting downstream of FGF2 signaling, is also required for mesoderm formation during early *Xenopus* development [97]. In mouse ESC studies, Yamaguchi et al. demonstrated that Fgf2 is involved in mesoderm patterning via FGF receptor type I [98]. However, in humans, the specific function of FGF2 in the commitment of mesodermal cells from hESCs to endothelial lineage is yet to be clarified [78]. Studies have demonstrated that FGF2 is required to maintain the undifferentiated state of hESCs, and its inhibition enhances BMP4-mediated differentiation towards the mesoderm [99,100,101]. While FGF2 and PI3K/Akt activity maintains self-renewal in hESCs, inhibition of PI3K/Akt signaling promotes the differentiation of mesoderm and endoderm [102]. Thus, the overall balance of canonical Wnt/β-catenin, activin/nodal, and BMP signaling needs be carefully controlled for early lineage specification during hiPSC-EC differentiation [103].

In efforts to efficiently differentiate ECs from hiPSCs through mesodermal lineage, combinations of such essential factors have been applied. In 2014, Orlova et al. improved the 2D mesoderm differentiation protocol generating functional ECs [104]. With treatments of BMP4, VEGF165, CHIR99021 and activin A for 3 days before replacing with endothelial inducing medium, the KDR^+^CD34^+^ mesodermal population showed an activin A-dependent increase (about 13%) by day 10. In the same year, Lian et al. successfully generated TBXT^+^ mesodermal cells from hiPSCs treated with CHIR99021 alone [105]. This suggested the possibility of lowering differentiation costs by using a single chemical inhibitor. In 2015, Patsch et al. attempted to identify chemical GSK3β inhibitors that promote hiPSCs toward mesoderm transition [76]. CHIR99021 or CP21R7 were the most effective, and in combination with BMP4 for Wnt activation resulted in a significant upregulation of genes associated with mesoderm, *TBXT, MIXL1,* and *EOMES*, and accumulation of TBXT expression levels, which peaked at day three. Patsch et al. confirmed that these GSK3β inhibitors with BMP4 and VEGFA in differentiation medium enhanced the efficiency exceeding 80%. Soon after, Liu et al. [106] specified that the size of hiPSC colonies, seeding densities of the colonies on Matrigel-coated dishes, and addition of CHIR99021 are critical with regard to mesoderm differentiation. Consistent with these findings, Lee et al. [73] demonstrated that transcriptional expression of *TBXT* and *KDR* was highly induced in conditions using collagen-coated surface and CHIR99021 treatment for three days. Enhanced hiPSC-mesodermal transition, resulting in more than 51% KDR^+^ population at this stage, enables more improved efficiency in EC differentiation. More recently, in 2020 Wang et al. used a condition of 48 h CHIR99021 treatment to convert hiPSCs into mesodermal progenitor cells and observed transient activation of mesodermal transcription factors *MIXL1* and *TBXT* [107].

Less commonly, studies have reported generation of hematopoietic/endothelial lineage progenitor cells from hiPSCs via CD34^+^ sorting. Supplemental BMP4 directed hESC differentiation towards both hematopoietic and endothelial cells [108,109]. Chadwick et al. [108] demonstrated that high levels of BMP4 enhanced hematopoietic stem cell survival, whereas Goldman et al. [109] reported that a short and high-dose boost of BMP4 in addition to VEGF accelerated CDH5^+^KDR^+^ endothelial commitment in vitro. In 2009, Choi et al. [110] obtained hiPSC-derived CD34^+^ cells that can differentiate into both hematopoietic and endothelial lineages. According to Yang et al. [111], CD34^+^ cells exhibited a more adherent endothelial progenitor-like phenotype under endothelial culture conditions, exhibiting the robust mRNA expression of endothelial markers *VWF*, *CDH5*, and *KDR*. Prasain et al. [112] optimized concentrations of activin A, BMP4, FGF2, and VEGF, critical for the emergence of mesoderm cells and thus ECs, compared to previous EC differentiation protocols. They generated hiPSC-derived intermediate cells positive for PECAM1 and NRP1, which give rise to CB-ECFC-like cells displaying a stable endothelial phenotype and possessing a high proliferative capacity. Double EC markers, CD34^+^PECAM1^+^, CD34^+^CDH5^+^, or CD34^+^KDR^+^ were used to isolate endothelial lineage cells. Moreover, Li et al. [113] used partially-induced hPSCs that transiently expressed only Oct4 and Klf4 for endothelial differentiation. They gradually changed supplements in the culture medium, exchanging BMP4 with 8-Br-cAMP in addition to VEGF and FGF2, thereby creating ‘induced’ ECs (iEnd cells). 

The second step for endothelial cell generation is to differentiate mesodermal lineage cells into ECs by various combinations of growth factors, cytokines, and small molecules. In addition to VEGF, the central factor of endothelial differentiation, hiPSC-derived ECs, are often generated by adding BMP4, FGF2, a TGF-β receptor I inhibitor (SB431542), a cAMP inducer (forskolin), and DLL4. VEGF acts as a key regulator of vasculogenesis and EC migration in angiogenesis [114]. VEGF signaling through VEGFR1 and VEGFR2 is transduced to mediate PI3K/Akt pathways. Mouse and zebrafish studies have shown that VEGF stimulates EC migration, merging into blood islands, and formation of the first primitive vascular plexus in the embryo [114]. In a hESC-derived EC study, VEGF plays an indispensable role for EC differentiation promoting expression of EC-specific proteins, although continuous treatment of VEGF was not able to enhance the rate of EC proliferation *per se* [115]. Genetic studies in mice revealed that BMP4 and FGF2 are not only critical for mesoderm formation but also play important roles in EC differentiation [78]. Mice deficient for BMP4 downstream effectors, such as *Smad5* or *Smad4*, lacked an organized yolk sac vasculature or displayed severe cell proliferation defects [116,117]. Ribatti et al. demonstrated that FGF2 signaling is important for in vivo formation of extraembryonic blood vessels [118]. FGF2 plays an important role in controlling endothelial homeostasis by maintaining endothelial fate. By inhibition of TGF-β signaling cascade, FGF2 prevents endothelial-mesenchymal transition and sustains endothelial features [119,120]. DLL4 has been shown to enhance the efficacy of EC differentiation but inhibit hematopoietic-lineage differentiation. Liu et al. [121] reported for the first time that VEGF can induce NOTCH1 and its ligand DLL4 in human arterial ECs. Small molecules such as SB431542, an inhibitor of TGF-β receptor I and or forskolin, a cAMP inducer, in combinations with VEGF can also promote efficient EC conversions from hPSCs [75]. 

Aiming for efficient and clinically applicable differentiation of hiPSC-derived ECs, optimization of EC-promoting media with defined conditions has been attempted. Wang et al. [122] differentiated hiPSC-derived ECs in medium containing VEGF, BMP4, FGF2 in the first stage, and SB431542 (TGF-β inhibitor) and Y27632 (ROCK inhibitor) in the next stage. Their protocol resulted in 60% purity and, within only one week, they achieved ECs exhibiting strong expression of the EC markers, PECAM1 and VWF, able to form capillary-like networks, and mimicking inflammatory responses of primary ECs. To induce endothelial lineage cells, Lee et al. [73] cultured hiPSC-derived mesodermal lineage cells in medium containing VEGF, FGF2, EGF, DLL4 and heparin and sorted the differentiated cells at day 14 with CDH5 antibody. Addition of DLL4 increased the PECAM1^+^, CDH5^+^, VWF^+^ cell population confirming that DLL4 further promotes EC differentiation. In 2017, Ikuno et al. [123] established that cAMP synergistically enhances VEGF effects in hiPSC-derived EC induction by supplementing with activin A, BMP4, FGF2, 8-Br-cAMP, and VEGF in the EC induction medium. By sorting with CDH5 and/or PECAM1 antibodies at 9 days after differentiation, they acquired an EC population at more than 99% purity. To perform large-scale single cell RNA sequencing, Paik et al. [124] generated hiPSC-derived ECs via mesodermal lineage differentiation. The cells were subjected to a commercial EC medium supplemented with BMP4, VEGF, and FGF2. They isolated CDH5^+^ cells on day 12 and further cultured in EGM2 medium supplemented with SB431542.

Recent studies have reported EC lineage differentiation from mesodermal cells via ETS variant 2 (ETV2) overexpression. It is well known that early induction of ETV2 expression is essential for embryonic vascular development. ETV2 is a member of the ETS family of transcription factors that is important for cell proliferation, differentiation, and migration [125]. These transcriptional regulators also play a critical role in EC development. The regulatory domains of many endothelial-specific genes contain ETS binding sites that include a core GGAA/T DNA sequence [126]. De Val et al. demonstrated that ETV2, in particular, regulates the differentiation of mesodermal progenitors toward hematopoietic and endothelial cell fate [127]. An enhanced level of expression of *Etv2* promotes expression levels of endothelial-specific genes, suggesting it is necessary and sufficient for endothelial cell development in vivo. In 2020, Wang et al. generated ectopic expression of *ETV2* by chemically modified mRNA (modRNA) for the mesoderm-to-endothelial differentiation stage [107]. Independent of exogenous VEGFA signaling, constant activation of synthetic *ETV2* in mesodermal lineage cells generated sufficient numbers of ECs in a rapid and robust manner [107]. It was shown that the differentiation efficiency was higher than 90%. Rather than activating endogenous *ETV2* expression by exogenous VEGF exposure, enhanced *ETV2* expression level was achieved by introducing modRNA to mesodermal cells via electroporation or lipofection-mediated methods. Indeed, studies reported that increased expression of *ETV2* in hiPSCs facilitates bypassing of the intermediate mesodermal stage to generate functional ECs [128,129,130], whereas most of the vascular differentiation approaches comprise sequential transitions. Interestingly, one recent study has revealed that transient expression of transcription factor *ETV2* is sufficient to directly reprogram somatic cells into ECs [131].

Endothelial specification for arterial, venous or lymphatic EC fates can be further induced in vitro, whereas in the developing embryo the primitive vascular plexus is specialized into arterial, venous or lymphatic-fated angioblasts [132,133]. In 2013, Rufaihah et al. [134] revealed that an hiPSC-derived EC population, purified for PECAM1, exhibited EC subtype heterogeneity, displaying arterial, venous and lymphatic lineage markers. Recently, Arora et al. [135] extensively reviewed heterogeneity within differentiated EC populations from PSCs and described phenotypic and functional differences between arterial and venous ECs. Moreover, through sequential EC maturation processes, arterial and venous-like ECs develop into functional blood vessels, such as arteries, veins and capillaries. In the late 1990s, Wang et al. [136] demonstrated that EFNB2 and its receptor EPHB4, mark arterial and venous ECs, respectively. Genetically arterial and venous ECs are distinguished by elevated expression of EFNB2, NRP1, NOTCH1, DLL4, and Jagged for arterial, and EPHB4, NRP2, and NR2F2 for venous ECs [135,136]. In 2014, Corada et al. [137] also reviewed signaling pathways, particularly Wnt, Sox, and Notch pathways, and transcription factors implicated in endothelial subtype specification. As a critical inducing agent, VEGF is not only required for EC proliferation by binding to KDR or VEGFR1, but also for specification of EC fates by binding to VEGFR2; with high and low concentrations leading to arterial and venous ECs, respectively [138,139,140]. Rosa et al. [141] succeeded in inducing hiPSC-derived arterial and venous-like ECs with chemically defined conditions via two-step differentiation protocols. Initially, hiPSCs were differentiated into endothelial precursor cells, marked as PECAM1^+^/KDR^+^/CDH5^med^/EPHB2^−^/NR2F2^−^. These cells were then directed into arterial or venous-like ECs by either high or low concentration of VEGF. Rosa et al. confirmed that hiPSC-derived arterial-like ECs produced more NO and elongated upon shear stress compared to hiPSC-derived venous-like ECs, consistent with a previous study conducted by Zhang et al. [142]. Further analysis is required to determine whether arterial or venous ECs are more effective for neovascularization.

### 2.2. Criteria to Define EC Identity

To validate the EC characteristics of hiPSC-derived ECs via various approaches, their molecular, cellular, and functional features must be carefully analyzed. Even though the differentiated cells can be purified with antibodies against EC-specific surface markers [69,73,76], it is essential to demonstrate key characteristics of ECs in hiPSC-derived ECs in a comprehensive manner. In this section, we summarize essential criteria for defining hiPSC-derived ECs (Table 1).

Conventionally, differentiated ECs are assessed for their morphology, EC-specific marker expression, and functionality in vitro. Microscopic observation is the primary technique to distinguish differentiated ECs from hiPSCs. The typical ‘cobblestone’ monolayer pattern of ECs should be seen at stationary density [143]. Molecular features of EC must be then addressed. Elevated expression of KDR, CDH5, VWF, PECAM1, TEK, and NOS3 at mRNA and protein levels are expected in hiPSC-derived ECs. Although human ECs are heterogenous among organs and tissues, the genetic profiles acquired by Marcu et al. [144] confirmed that general EC markers, such as *PECAM1*, *CDH5*, and *VWF* are commonly expressed across organs including heart, lung, liver and kidney. Protein expression levels and localization of such markers in hiPSC-derived ECs need to be demonstrated by Western blotting and immunostaining methods. Differentiated hiPSC-ECs should display CDH5 and KDR on the cell surface, CDH5 and PECAM1 at cell junctions, VWF and ANGPT2 in Weibel-Palade bodies (endothelial restricted organelles), and NOS3 at perinuclear regions and the plasma membrane [145]. Nevertheless, expression levels of these markers may be altered depending on the maturity of hiPSC-derived ECs. For example, KDR expression is sustained in young endothelium [131], whereas it had been often used as a marker for mesodermal lineage progenitors [69,73,104]. Mature hiPSC-derived ECs may display different protein profiles compared to immature ones. For instance, White et al. demonstrated that hiPSC-derived endothelial progenitor cells expressing only KDR were further differentiated into more mature ECs expressing CDH5 and PECAM1 [146]. Patel et al. revealed that PECAM1 is a marker for more mature, but not young, ECs [147]. Likewise, Adams et al. revealed that a KDR^+^PECAM1^+^ double positive population of hiPSC-derived ECs lacked CDH5 expression while a subpopulation of PECAM1^+^ cells may also express the hematopoietic marker CD45 [145]. Thus, we suggest that combinations of EC markers be used to support endothelial differentiation from hiPSCs. In addition, functional abilities of hiPSC-derived ECs, such as NO production and AcLDL uptake, should be assessed. NO production is certainly one of the most crucial characteristics of ECs. Constant release of NO, encoded by endothelial-specific NOS3, controls vessel tone [148,149] and stimulates cell migration and angiogenesis [114]. Thus, quantification of NOS3 expression at both the mRNA and protein levels should be documented from hiPSC-derived ECs. Indeed, NO release could be easily measured by fluorescent bioimaging techniques [150,151,152] that quantify nitrate-to-nitrite conversion. Also, DiI-labeled AcLDL is frequently used as an endothelial marker. Angiogenic properties of hiPSC-derived ECs can be included, as hiPSC-ECs are enriched with proangiogenic factors. Expression of proangiogenic factors such as VEGFA, IGF1, ANGPT1 and FGF2 can be measured at the molecular level by RT-PCR or ELISA [50]. Cell migration assay and tube-like network formation assay are essential, because in the native environment during angiogenesis and sprouting, ECs are stimulated to migrate, forming new capillaries. In a cellular migration assay, cell migration toward a cell-free gap in a monolayered culture is quantified in comparison with other cell types [153]. Tube formation ability of hiPSC-derived ECs is often analyzed on a Matrigel 3D matrix [154]. The number of branching points and branch lengths are measured. In silico analyses of hiPSC-EC transcriptome via RNA sequencing can also be performed. Paik et al. utilized a droplet-based method to determine single-cell RNA sequencing of hiPSC-derived ECs [124]. Based on sequencing data analysis, their hiPSC-EC differentiation protocol produced a large population of iPSC-ECs but also other cell types of mesodermal lineages. They discovered that the generated hiPSC-ECs population were predominantly arterial-like ECs, with low numbers of venous and lymphatic ECs.

## 3. Human Induced Pluripotent Stem Cell-Derived Smooth Muscle Cells

A number of methods have been developed to differentiate hiPSCs into vascular SMCs. The use of EB, which mimics early embryonic development in mammals, provided the opportunity to directly differentiate into SMCs [155,156,157,158,159]; however, owing to the line-to-line variability in efficiency and labor-intensive nature of EB-based protocols, the monolayer-based protocols have been favored. Accordingly, in this section, we review recent progress in monolayer-based differentiation systems of hiPSCs towards SMCs.

### 3.1. Differentiation of hiPSCs into Lineage-Specific SMC Intermediates

**Figure 3:**
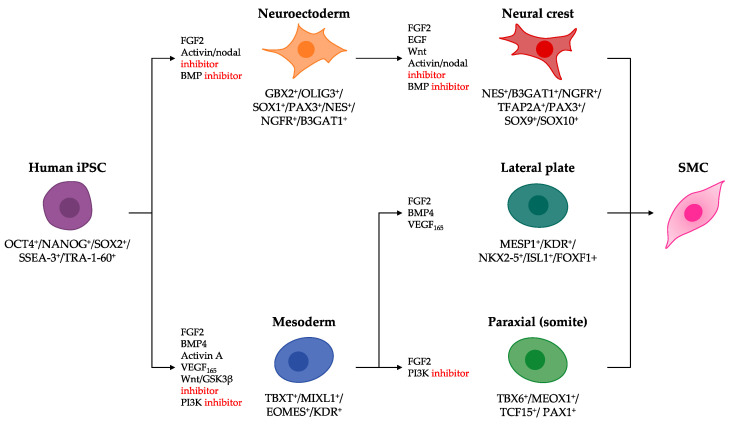
Proposed strategies to derive lineage-specific SMC intermediates from hiPSCs. Derivation of lineage-specific SMC intermediates from hiPSCs recapitulates the defining events of early embryonic development and cell lineage specification in vertebrate embryos. Combinations of growth factors, cytokines and phenotypic markers that define each cell type are listed.

Before considering any method for differentiation of hiPSCs into SMCs, it is essential to note that vascular SMCs arise from distinct developmental origins (reviewed in detail by Majesky [160], and also by Sinha et al. [161]). For example, a fraction of vascular SMCs are neuroectodermally derived neural crest cells, while most originate from the mesodermal lineages, including the lateral plate and paraxial mesoderm [162,163,164]. Accordingly, this principle was extended to generate hiPSC-derived lineage-specific SMC intermediates before differentiating further into vascular SMCs (Figure 3). In the same vein as the developmental process from which hiPSC-derived ECs emerge, several strategies to derive lineage-specific SMC intermediates from hiPSCs also depend on key factors involved in embryonic vascular development.

Studies have shown that hiPSCs could be differentiated into vascular SMCs through the neural crest lineage. In general, different combinations of the FGF [165,166,167], BMP [168,169,170], Wnt [171,172], TGF-β [173,174], RA [175,176], and/or activin/nodal [177,178] signals were utilized and optimized to differentiate into a neural crest lineage endowed with SMC potential [179]. Wang et al. [180] introduced a protocol to allow derivation of neural crest stem cells from hiPSCs before differentiating into SMCs. Their method was sufficient to give rise to NES^+^/B3GAT1^+^/NGFR^+^/TFAP2A^+^ cells by using a publicly available neural supplement with FGF2 and EGF. However, one caveat in this study was that their neural rosette-based method was time-consuming, taking at least 22 days, and obtained a relatively low yield. In 2012 and 2014, Cheung et al. [181,182,183] provided a detailed characterization of neural crest cells derived from hiPSCs. The former adopted their previously published protocol [184] using a chemically defined medium supplemented with FGF2 and activin/nodal inhibitor SB431542, while the latter was a combination of FGF2, SB431542, and a BMP inhibitor, noggin [185], resulting in a substantial amount of *GBX2*^+^/*OLIG3*^+^/*SOX1*^+^ neuroectodermal cells. In addition, PAX3 and NES were observed in these cells by immunocytochemistry. The differentiated cells were then isolated by FACS with antibodies against NGFR and B3GAT1. Enriched gene expression of *PAX3*, *NGFR*, *B3GAT1*, and *TFAP2A* was detected in the sorted cells. It is noteworthy that the concept of systemically deriving three lineage-specific SMC intermediates from a single hiPSC in vitro was first reported by Cheung et al. [181], as studies from a number of different groups have only featured the generation of a single lineage-specific SMC intermediate from hiPSCs, mainly of the mesodermal lineage. Based on the results from previous studies [181,186], Halaidych et al. [187] found that a combination of FGF2 and SB431542, with a small molecular Wnt activator, CHIR99021, was the most efficient in induction of the neural crest lineage. FACS analysis on day 12 of differentiation showed that approximately 40–50% of cells were positive for NGFR and B3GAT but negative for TRA-1-60 and SOX2. This cell population was further enriched by mechanical removal of undifferentiated colonies, and expressed the increased gene expression of *TFAP2A*, *SOX9* and *10*, and *PAX3*. Their protocol was highly reproducible in the three different hiPSC lines. These studies show that careful control over the inductive and inhibitory signals should be exercised for specification of the neural crest.

As vascular SMCs mostly originate from the mesoderm during development, many protocols for generating vascular SMCs from hiPSCs include a mesodermal step. Developmentally, canonical Wnt signaling is indispensable for the mesoderm commitment [85,188]. For one, mice carrying a null mutation in Wnt signaling display defects in mesoderm formation [82,189,190]. Moreover, Wnt signals act in concert with activin/nodal and BMP signaling to specify mesodermal cell fate [103,191]. Each morphogen can elicit mesodermal responses with a tightly controlled concentration gradient, whereas the members of the FGF family do so over relatively broad ranges [192]. Such spatiotemporal presentation inevitably translates into distinct mesoderm subtypes [193]. For example, Cheung et al. [181] demonstrated the feasibility of deriving two lineage-specific SMC mesodermal intermediates from hiPSCs: lateral plate and paraxial mesoderm. Human iPSCs were initially differentiated into early mesoderm using a combination of FGF2, BMP4, and a PI3K inhibitor in which a primitive streak [194,195] and early mesoderm marker [196], *TBXT*, emerged and peaked at 36 h. For further specification to the lateral plate mesoderm, only FGF2 and BMP4 were added to upregulate gene expression of lateral plate mesoderm markers, including *MESP1*, *KDR*, *NKX2*-5, and *ISL1*. In contrast, a combination of only FGF2 and a PI3K inhibitor increased the gene expression of paraxial mesoderm markers, including *TBX6*, *MEOX1*, *TCF15*, and *PAX1*. Concomitant protein expression of lateral plate (KDR, ISL1, NKX2-5) and paraxial mesoderm markers (TCF15 and TBX6) was observed under these two conditions. Their method gave similar results in subsequent studies from Iyer et al. [197], and Granata et al. [198]. To differentiate lateral plate mesodermal cells into the epicardial lineage, Iyer et al. revealed that modulation of WNT3A, BMP4, and RA signals is responsible. A strategy developed by Patsch et al. was to identify selective GSK3β inhibitors that promote canonical Wnt signaling to direct the differentiation of hiPSCs into mesodermal cells [76]. It was shown that CHIR99021 and a commercially available compound, CP21R7, were the most efficient in induction of the mesodermal lineage, and concerted treatment with BMP4 enhanced its differentiation efficiency. Treatment of hiPSCs with CHIR99021 and BMP4 led to upregulation of *TBXT*, *MIXL*, and *EOMES*; conversely, these TBXT^+^ cells showed a significant reduction in the expression of POU5F1. Similar results were obtained using CP21R7 and BMP4. Using the same recipe as in a publicly available B27 supplement, Yang et al. [199] directed hiPSCs towards the mesodermal lineage. However, in vitro characterization of hiPSC-derived mesodermal cells was not investigated in this study. Kwong et al. [200] provided a novel approach to differentiate hiPSCs into the mesodermal lineage, which was adapted from a previously published protocol from Prasain et al. [112]. Human iPSCs were first cultured in a serum-free medium supplemented with activin A, FGF2, BMP4, and VEGF_165_ for a day, and then the medium was replaced with a commercially available hematopoietic stem cell expansion medium containing FGF2, BMP4, and VEGF_165_ for three days. At day four, Kwong et al. identified a population of putative mesodermal cells with robust KDR expression, which was also enriched for a lateral plate mesoderm marker, *FOXF1*. Synergistic combinations of key developmental signals can program hiPSCs to differentiate into the mesodermal lineage.

While there is a plethora of cell culture methodologies to differentiate hiPSCs to the mesodermal lineage, many have attempted to obtain hiPSC-derived vascular SMCs from other SMC intermediates. Intriguingly, groups of investigators first differentiated hiPSCs into CD34^+^ (with PECAM1^+^) progenitor cells [105,201,202,203]. For example, Lian et al. [105] and Bao et al. [203] showed that a combination of ascorbic acid and CHIR99021 was sufficient to induce a population of CD34^+^/PECAM1^+^ progenitor cells. Under alternate differentiation conditions, sorted cells could give rise to both ECs and SMCs. In 2012, Bajpai et al. [204] provided a stepwise process that began with a epithelial-to-mesenchymal transition from hiPSCs to highly proliferative and multipotent mesenchymal stem cells (MSCs), not mesodermal cells, in a commercially available human vascular SMC basal medium containing SM differentiation supplement with 5% FBS, FGF2, EGF, heparin, and insulin. Human iPSC-derived MSCs acquired the characteristics of MSCs, expressing high levels of NT5E, THY1, ITGA2, CD44, and ENG. The resulting cells displayed differentiation potential to obtain an osteogenic, adipogenic, and/or chondrogenic phenotype, before specifying towards a SMC lineage. Innovatively, Kumar et al. [205] proposed that mesodermal pericytes and SMCs derived from hiPSCs originate from an endothelial and mesenchymal cell precursor, the mesenchymoangioblast. Initially employed in an hESC differentiation system in 2010 [206], Kumar et al. differentiated hiPSCs into a mesodermal cell population with BMP4, activin A, and FGF2, using the cocktail developed by Uenishi et al. [207]. The APLNR^+^/PDGFRα^+^ mesodermal cells were then molded into FGF2-dependent compact spheroid colonies that exhibited both mesenchymal and endothelial potentials. These colonies further morphed into mesenchymoangioblasts expressing NGFR^+^/ENG^+^/PDGFRβ^+^/MCAM^+^/NT5E^−^. Mesenchymoangioblasts reminiscent of lateral plate mesoderm-derived embryonic mesenchyme later gave rise to either pericytes or SMCs. Collectively, these strategies for inducing differentiation of hiPSCs to nonspecific and lineage-specific SMC intermediates offer valuable insights into the developmental pathways that are crucial for vascular SMC biology.

### 3.2. Differentiation of hiPSCs into Specialized SMC Phenotypes

**Figure 4:**
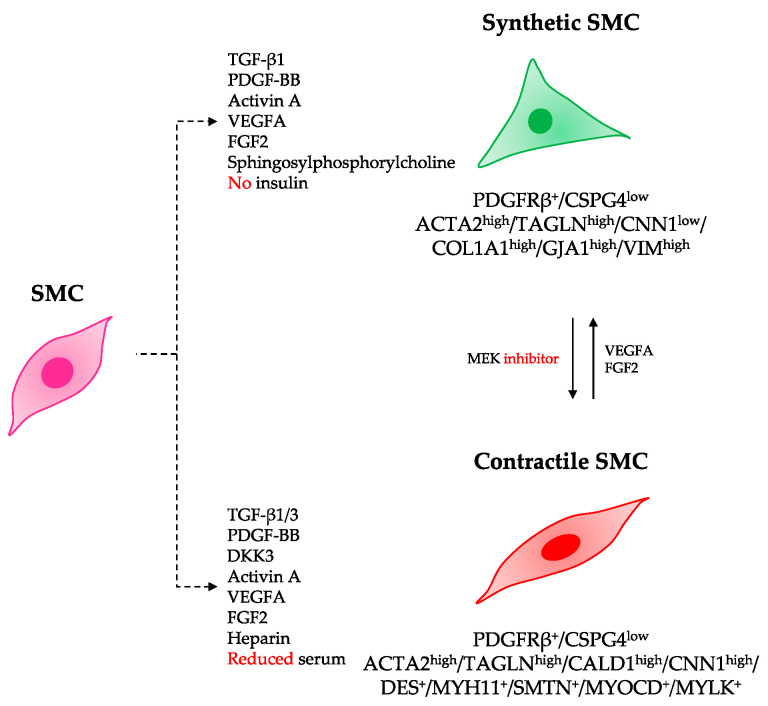
Proposed strategies to derive synthetic and contractile SMCs from hiPSCs. Differentiation of hiPSCs into specialized SMC phenotypes can be modulated by combinations of growth factors and cytokines, and by concentration of serum. Phenotypic markers that define each phenotype are listed.

Unlike either skeletal or cardiac muscle cells, SMCs retain a remarkable phenotypic plasticity, reversibly shifting along a continuum from a quiescent, contractile phenotype to a proliferative, synthetic phenotype (reviewed in detail by Owens et al. [208]) (Figure 4). Thus, a similar but distinct differentiation method must be applied to derive each phenotype from hiPSCs.

Taura et al. [69] were the first to generate mural cells from the four hiPSC cell lines. Although differentiation efficiency was low, Taura et al. used a protocol that was previously established for hESCs [43] to sort for a population of KDR^+^/CDH5^−^/TRA-1-60^−^ cells. Sorted cells were further differentiated into mural cells using only PDGF-BB. These PDGF-BB-treated cells expressed ACTA2^+^/CNN1^+^, reminiscent of vascular SMCs. Similarly, Patsch et al. [76] treated mesodermal cells with PDGF-BB and activin A in a mixture of two commercially available supplements, N2 and B27 (N2B27), for two days. The resulting cells expressed PDGFRβ^+^/CDH5^−^ and were cultured in N2B27 medium supplemented with PDGF-BB for another five to seven days to obtain hiPSC-derived synthetic SMCs. Alternatively, to promote a contractile SMC phenotype, the treated cells were cultured in N2B27 medium supplemented with activin A and heparin. The resulting cells also expressed ACTA2, TAGLN, and MYH11b, and exhibited a transcriptional signature and metabolic profile similar to human pulmonary and aortic vascular SMCs. Differentiated SMCs were functional, as these cells were capable of responding to vasoconstrictive stimuli as shown by calcium imaging and a collagen gel contraction assay, demonstrating their contractility. Lastly, the differentiated SMCs deposited extracellular fibronectin, confirming their functionality [209]. However, Patsch et al. routinely used both human ESC and iPSC lines, which were denoted as hPSCs; thus, there was no indication as to which hPSC line was employed. Lastly, Karamariti et al. [210] cultured partial-iPSCs with PDGF-BB for six days, as previously developed by Xiao et al. [211]. The differentiated partial-iPSCs expressed a repertoire of contractile SMC-specific markers (*ACTA2*, *TAGLN*, *CNN1*, *MYH11*, *SMTN*, *MYOCD*, and *SRF*) at the mRNA level, and ACTA2, TAGLN, and CNN1 at the protein level. Immunocytochemistry for TAGLN and CNN1 demonstrated a SMC-like morphology in the differentiated partial-iPSCs. Stimulation of partial-iPSC-derived SMCs with KCl caused a strong contraction, which was similar to human SMCs. Interestingly, treatment with a member of DKK3 demonstrated an upregulation of SMC-specific marker expression in the DKK3-treated cells at both the mRNA and protein levels. Groups of investigators demonstrated the importance of PDGF-β/PDGFR-β signaling in mural cell specification, as signaling is required for vascular integrity during blood vessel development [212,213,214].

TGF-β signaling is implicated in the development of the contractile SMC phenotype [215,216,217]. Consequently, Wang et al. [180] further derived SMCs of neural crest origin using 5% FBS and TGF-β1 only. After two weeks of culture, these cells showed the assembly of ACTA2, TAGLN, and CNN1 into stress fibers, but with diffuse cytoplasmic staining of MYH11. In addition, Bajpai et al. [204] tested different combinations and found that a combination of TGF-β1 and heparin induced relatively higher gene expression of *ACTA2*, *TAGLN*, *CALD1*, *CNN1*, and *MYH11* in hiPSC-derived SMCs. Similarly, immunocytochemistry showed a filamentous organization of ACTA2, CNN1, and MYH11 in these cells. One defining property of mature SMCs is to produce force in response to vasoactive agonists. Bajpai et al. thus fabricated small-diameter cylindrical tissue constructs by embedding hiPSC-derived SMCs in fibrin hydrogels. After two weeks of culture in medium containing TGF-β1, insulin, and ascorbic acid, the tissue constructs, which were distributed uniformly and circumferentially with the hiPSC-SMCs, exhibited robust force generation in response to vasoactive agonists, and superior mechanical strength. Lastly, Kumar et al. [205] used TGF-β1 and sphingosylphosphorylcholine together to produce hiPSC-derived SMCs and provided detailed morphological and functional characteristics of immature and mature hiPSC-derived SMCs. Human iPSC-derived SMCs became much larger and acquired the typical rhomboid morphology of synthetic SMCs. Although the cells showed increased expression of ACTA2 and CNN1, a population of highly proliferative and expandable cells were designated as immature SMCs. Based on a finding that a MAPK1 cascade plays a critical role in the regulation of protein synthesis in SMCs [218], Kumar et al. treated immature hiPSC-SMCs with the MAP2K7 inhibitor PD0325901, whereby they acquired a more elongated morphology and expressed a mature SMC marker, MYH11. There was a substantial increase in the expression of other SMC markers, ACTA2, DES, and CNN1, in mature hiPSC-SMCs. In addition, gene expression profiling revealed a unique molecular signature for transition from immature to mature hiPSC-derived SMCs. A significant upregulation of typical SMC genes, including *ACTA2*, *TAGLN*, *CNN1*, *MYH11*, *MYOCD*, *MYLK*, *LMOD1*, and *SYNPO2*, and genes enriched in the KEGG categories, including focal adhesion and vascular SMC contraction, was consistent with the maturation of SMCs. To determine the contractile properties of hiPSC-derived SMCs, Kumar et al. performed time-lapse studies of individual cells treated with carbachol. Mature hiPSC-SMCs responded to carbachol treatment much more strongly and contracted in a tonic fashion. Similarly, these mature cells exhibited the strongest basal contractile tone in the gel lattice assay, and to a much lesser degree in immature hiPSC-derived SMCs. Finally, a Matrigel plug assay showed the capacity of hiPSC-derived SMCs to support vasculature formation in vivo. The results suggested that TGF-β signaling might be required to induce differentiation of mature and contractile SMCs.

As both PDGF-BB and TGF-β1 play crucial roles in induction of a vascular SMC phenotype, Cheung et al. tested the two to differentiate three different lineage-specific SMC intermediates from hiPSCs into contractile SMCs [181,182,183]. Treatment with PDGF-BB and TGF-β1 promoted the elevated gene expression of early-intermediate (*ACTA2*, *TAGLN*, and *CNN1*) and late (*MYH11* and *SMTN*) SMC markers at day 12. Flow cytometric analysis correlated with the gene expression profile, generating more than 80% ACTA2^+^/MYH11^+^ and TAGLN^+^/MYH11^+^ cells. Immunocytochemistry demonstrated extensive staining for CNN1 and TAGLN in the derived SMCs. Western blot also revealed an increase in the protein expression of mature SMC markers MYH11 and SMTN in the derived SMCs. A microarray analysis was conducted to characterize the derived SMCs, showing upregulation of vascular SMC genes. Cheung et al. [181] further assessed their contractile potential by first detecting a change in intracellular calcium flux and then by measuring a change in surface area of cell size in response to carbachol treatment. As a result, hiPSC-derived SMCs possess contractile function. To determine whether these cells could contribute to vessel formation in vivo, a Matrigel plug of mixed human umbilical cord vein endothelial cells (HUVEC) and hiPSC-derived SMCs was subcutaneously implanted into immunodeficient mice for two weeks. Human iPSC-derived SMCs stained for SMTN were recruited to peri-endothelial regions. A complete in vitro characterization of hiPSC-derived SMCs was provided by Cheung et al. [181,183]. Halaidych et al. [187] differentiated neural crest cells derived from hiPSCs into vascular SMCs using previously described protocols [181,198,219,220,221], with some modifications. There were five modified protocols involved with different combinations of TGF-β3, PDGF-BB, and FBS. Gene expression of contractile SMC markers, including *ACTA2*, *TAGLN*, *CNN1*, and *MYH11*, was similar in hiPSC-derived SMCs from the five differentiation protocols. Likewise, a contractile phenotype was confirmed by immunocytochemistry for ACTA2, TAGLN, and CNN1. Halaidych et al. further demonstrated subpopulations of hiPSC-derived SMCs with varying intracellular calcium release and reuptake, but mostly with strong contractions in response to a vasoconstrictor. Kwong et al. [200] also used a combination of TGF-β1 and PDGF-BB to directly differentiate into hiPSC-derived SMCs. Kwong et al. sought to engineer putative SMCs from hiPSCs by targeting an eGFP reporter to the endogenous human ACTA2 locus. At day 30, about 36% of ACTA2^eGFP+^ cells were present, and the sorted cells expressed the characteristics of vascular SMCs in which *ACTA2*, *TAGLN*, and *MYH11* were increased at the mRNA level. A transcriptomic profile of the ACTA2^eGFP+^ and ACTA2^eGFP-^ cells verified that the ACTA2^eGFP+^ cell population was suggestive of an immature and/or synthetic SMC phenotype. Lastly, Yang et al. [199] presented two protocols for differentiating into either a synthetic or contractile SMC phenotype of the mesodermal lineage. To differentiate into a synthetic SMC phenotype, the mesodermal cells were treated with different combinations of (1) VEGFA and FGF2 in a publicly available B27 supplement minus insulin for four days; (2) VEGFA and FGF2 in a B27 supplement for two days, and (3) PDGF-BB and TGF-β1 in a B27 supplement for four days. To differentiate into a contractile SMC phenotype, the mesodermal cells were treated with combination (1) for four days, and with combination (3) for six days. The differentiated cells were then enriched for SMCs in a medium containing lactate for four to six days. While the purified cells from each differentiation protocol showed more than 90% of ACTA2^+^ cells, hiPSC-derived contractile SMCs showed the relatively higher gene expression of *MYH11*, *CNN1*, and *TAGLN* than did synthetic SMCs. However, hiPSC-derived synthetic SMCs were more likely to express COL1A1, GJA1, and VIM. Immunocytochemistry demonstrated much higher protein expression of CNN1 in hiPSC-derived contractile SMCs. Differences in their functionality were detected. Human iPSC-derived synthetic SMCs were highly migratory and proliferative, whereas contractile SMCs were more contracted in response to carbachol treatment. The size of fibrinogen gels suspended in each cell type was measured after three days. Gels containing hiPSC-derived contractile SMCs contracted to approximately 27% of their original size, compared to 46% for gels containing synthetic SMCs. Yang et al. utilized a metabolic selection to purify the hiPSC-derived contractile SMCs. Two major factors, PDGF-BB and TGF, are frequently used to specify towards SMC phenotypes. More faithful recapitulation of the developmental pathways that undergo SMC specifications may represent an efficient route to improve the differentiation yield and purity.

### 3.3. Criteria to Define the Contractile SMC Identity

In all methods for differentiation of hiPSCs, it is imperative to define the key characteristics of the desired cell type, such as ECs and cardiomyocytes. However, this task has proven particularly daunting for hiPSC-derived SMCs, as their heterogeneity in origin contributes to the varying functions and expression of SMC-specific markers [222,223,224,225]. Phenotypic switching of vascular SMCs, which constantly fluctuate between a proliferative, migratory, ECM-producing, immature, and synthetic phenotype and a quiescent, mature, and contractile phenotype, further adds to the SMC heterogeneity (reviewed in detail by Owens et al. [208]). As such, a clear understanding of what constitutes mature and contractile SMCs is desperately needed. Differentiation efficiency can, therefore, be reported by means of (1) flow cytometric analysis for contractile SMC-specific markers, (2) quantitative analysis using SMC-specific fluorescent reporter hiPSC lines, and/or (3) quantitative analysis of intracellular calcium release and contraction in hiPSC-derived SMCs. In this section, we highlight the characteristics that must be present to consider a cell a mature and contractile SMC (Table 2).

Examination of the cell morphology is a major indicator to define cell identify. Nonetheless, morphological differences in SMCs are often observed because of their heterogeneity and phenotypic switching. Typically, contractile SMCs are elongated and spindle-shaped, whereas synthetic SMCs are epithelioid or rhomboid-shaped [226]. Electron microscopy more accurately reflects the ultrastructural features of the hiPSC-SMCs. The arrangement of cytoplasmic dense bodies and their relationship to the filaments should be detected. As a general consensus holds that there is no single marker exclusively definitive for a mature and contractile SMC phenotype, hiPSC-derived SMCs should express a collection of contractile SMC-specific markers such as ACTA2, TAGLN, CALD1, DES, CNN1, MYH11, and SMTN at the mRNA and protein levels [208]. To date, the two markers that best define a mature and contractile SMC phenotype are MYH11 and SMTN. Immunocytochemistry for these proteins should reveal proper structural organization of thick and thin filaments. Downregulation of pluripotency markers, such as TRA-1-60, SSEA-3, POU5F1, and SOX2, should be apparent in hiPSC-derived SMCs. Although not necessarily required, expression of SMC fate-determining transcription factors that govern the transition to a contractile SMC phenotype can be determined. They are SRF [227], MYOCD [228,229,230], MRTFA and B [231], CRIP1 and 2 [232], and PITX2 [233]. Furthermore, a global analysis of gene expression using microarray, RNA-sequencing, or even single-cell RNA-sequencing, can be performed to further determine whether hiPSC-derived SMCs bear a strong resemblance to human contractile SMCs. The most reliable feature that a bona fide contractile SMC can show is its contractibility. The two commonly used functional assays, which are based on live-cell imaging, are (1) quantitative analysis of intracellular calcium release, and (2) contraction in response to vasoactive agonists. For the former, live human iPSC-derived SMCs are initially preloaded with a calcium-sensitive fluorescent dye and treated with vasoconstrictors to measure the oscillation of intracellular calcium concentrations by fluorescence imaging. For the latter, live human iPSC-derived SMCs are simply stimulated with a vasoconstrictor, and a series of images are acquired to calculate changes in the surface area of individual cells. The functionality of hiPSC-derived SMCs should then be compared with that of human SMCs. Recently, Halaidych et al. [187] provided open-source software for automated and unbiased analysis of intracellular calcium release kinetics and contractions by tracking individual cells. Thus, functional assessment of hiPSC-derived SMCs is of particular importance to distinguish between a contractile and noncontractile SMC phenotype. Taken together, contractile SMCs derived from hiPSCs should manifest all of the expected characteristics and functions, expressing contractile SMC markers and contracting in response to vasoconstrictors.

## 4. Therapeutic Applications Using hiPSC-Derived Vascular Cells

In this section, we discuss the feasibility and efficacy of hiPSC-derived vascular cells in animal models of experimental myocardial infarction and hindlimb ischemia before these cells can be applied to clinical application (Table 3).

Rufaihah et al. [70] were the first to demonstrate the therapeutic potential of hiPSC-derived ECs in a mouse model of hindlimb ischemia. When delivered into ischemic hindlimbs, hiPSC-derived ECs improved blood perfusion as seen by laser Doppler perfusion imaging and increased blood capillary density. Rufaihah et al. showed that injected hiPSC-derived ECs, mostly located in close proximity with only a few cells incorporated into the microvasculature, survived only up to 14 days. In concordance with these results, Clayton et al. [234] reported that perfusion recovery and capillary density were enhanced in the hiPSC-derived EC-injected ischemic tissues up to 14 days. Some transplanted hiPSC-derived ECs that were transduced with a GFP reporter construct were colocalized with PECAM1^+^ capillaries, indicating incorporation of these cells into the microvasculature. Although the two studies showed the regenerative effects of hiPSC-derived ECs in a mouse model of hindlimb ischemia, Rufaihah et al. [70] and Clayton et al. [234] only looked at their short-term in vivo behavior. Similarly, Lai et al. [235] intramuscularly injected hiPSC-derived ECs into three sites of ischemia hindlimbs and performed serial laser Doppler perfusion imaging up to 28 days. Lai et al. revealed that implantation of hiPSC-derived ECs enhanced blood flow and capillary density, which attenuated severe hindlimb ischemia in mice. However, their study was limited since they did not monitor the fate of transplanted hiPSC-derived ECs but only the similarities shared by generated ECs from different sources, including hiPSC-EC, hESC-EC, hBM-EC and HUVEC. Thus, the precise contribution of the hiPSC-derived ECs to neovascularization remained unknown. As these investigators showed low cell viability and survival after 28 days in a mouse model of hindlimb ischemia, the use of biomaterials such as hydrogels [236,237] and a nanomatrix gel [73] during cell delivery was applied in subsequent studies. Mulyasasmita et al. [236] developed a codelivery system of hiPSC-derived ECs with VEGF in a MICTH-PEG hydrogel, which not only provided significant protection from cell damage but also improved tissue regeneration and reduced inflammation in a mouse model of hindlimb ischemia. Foster et al. [237] also showed enhanced hiPSC-EC retention in vivo and improved neovascularization of the ischemic limbs following the delivery of hiPSC-derived ECs in a hybrid hydrogel called SHIELD. Biomaterial-encapsulated hiPSC-derived EC delivery appeared to improve the survival of implanted hiPSC-derived ECs, which allowed these transplanted cells to exhibit their regenerative effects in vivo. Lee et al. [73] were the only researchers to demonstrate that hiPSC-derived ECs encapsulated in peptide amphiphile-nanomatrix gel survived for more than ten months in the ischemic environment in vivo and improved therapeutic neovascularization. At the histological level, Lee et al. showed that a large population of these hiPSC-derived ECs, which possessed regenerative capacity, were incorporated into the host vessels, and contributed to new vessel formation. Additionally, iPSC-derived ECs have been evaluated in animal models of experimental myocardial infraction. Gu et al. [238] successfully generated porcine iPSC-derived ECs and showed their therapeutic potential to treat myocardial infarction in a porcine model. Collectively, the therapeutic potential of hiPSC-derived ECs in vivo is well-established.

Similarly, direct contribution of transplanted hiPSC-derived SMCs to neovascularization was assessed in animal models of ischemic cardiovascular diseases. Dar et al. [239] used a murine model of hindlimb ischemia to determine whether hiPSC-derived pericytes contribute to neovascularization. In addition to an accelerated recovery of perfusion, the vessel density was significantly higher in the hiPSC-derived pericyte-injected ischemic limbs. Histological analysis showed that fluorescent-labeled differentiated cells not only retained their perivascular features in vivo but also incorporated into host blood vessels, indicating their putative therapeutic role in regenerative medicine. Recently, Gao et al. [240] determined the therapeutic potential of hiPSC-derived SMCs in a murine model of hindlimb ischemia. Human iPSC-derived SMCs were intramuscularly injected at the sites of ischemia and ameliorated ischemia. Laser Doppler perfusion images demonstrated improved blood flow in the differentiated cell-injected mice, and histological findings correlated with functional outcomes, which had an increase in the fibrosis area and capillary density, and number of arterioles. Expression of VEGFA was significantly elevated with transplantation of hiPSC-derived SMCs, promoting VEGF-mediated angiogenesis. The results indicate that hiPSC-derived SMCs are a promising source to treat cardiovascular diseases. While efforts were recently made to evaluate the regenerative effects of hiPSC-derived SMCs on animal models of ischemic cardiovascular diseases, more studies are certainly needed.

While this is not the major focus of this review, hiPSC-derived vascular cells have also been utilized as a cell source for vascular tissue engineering. Specifically, tissue-engineered vascular grafts (TEVGs) using hiPSC-derived SMCs have been developed to serve as an innovative option to replace diseased blood vessels [158,200,204,210,219,241]. To date, TEVGs using hiPSC-derived SMCs have demonstrated significant mechanical strength and implantability [242]. Further studies should be directed to engineer a vascularized and permeable yet durable tissue graft, potentially transferring towards clinical applications.

## 5. Challenges and Future Directions

While the therapeutic potential of hiPSC-derived vascular cells has been demonstrated in animal models of myocardial infarction and hindlimb ischemia, several challenges must be overcome prior to their clinical translation. One major challenge using hiPSCs for cell therapy is the heterogeneity (genetic variability) of hiPSC lines and their derivatives. According to a review by Liang and Zhang [243], there are considerable genetic and epigenetic variations between the hiPSC lines and even between different passages of the same line. Such variations potentially influence their inherent pluripotency and differentiation capacity. This risk can be eliminated to a certain degree by using non-integrating vectors. For example, Hu et al. [244] established an integration-free hiPSC line from patient peripheral blood mononuclear cells through episomal vector nucleofection of the Yamanaka factors, which they successfully differentiated into functional SMCs. However, to minimize the associated risk, extensive genomic and epigenomic profiling should be conducted to exclude any lines with accumulated mutations.

Another concern is clinical compatibility of hiPSC-derived vascular cells. This embodies the following features: (1) establishment of fully defined and xenogeneic-free conditions to maintain the hiPSC lines and to differentiate into vascular cells; (2) optimization of protocols, which results in a highly reproducible differentiation efficiency and preferentially reduces cost and culture duration; (3) extensive in vitro characterization of hiPSC-derived vascular cells; and (4) isolation and/or purification of targeted vascular cells. First, hiPSCs must be differentiated in conditions that are not only fully defined but also free of xenogeneic components, as the use of animal serum and/or animal-derived feeder cells precludes clinical applications. One recent study by Luo et al. [245] established an efficient method for deriving functional hiPSC-derived SMCs under xenogeneic-free conditions. In addition, clinically compatible differentiation systems should be optimized to produce target cells with a highly reproducible efficiency. Then, in vitro characterization of the hiPSC lines and their derivatives should be provided to ensure their cell identity. Moreover, for a successful clinical translation of cell-based therapy using hiPSCs, a reliable and efficient method to purify or sort only target cells while preserving cell viability is a critical step. Although antibodies against surface makers, such as CDH5 [69,73,76], and PECAM1 [104,246], have been used for purification of hiPSC-derived ECs, there are as yet no applicable antibodies to target only hiPSC-derived SMCs. Yang et al. [199] used a metabolic selection to purify hiPSC-derived contractile SMCs; however, changes in metabolism may influence stemness and cell fate specification [247,248]. Thus, a novel method must be developed to obtain a homogenous population of hiPSC-derived contractile SMCs. Collectively, meticulous planning is extremely important to produce clinically compatible systems to derive functional vascular cells from various hiPSC lines. 

Although many of the limitations indicated above have been addressed for hiPSC-derived ECs such that they are now considered an emerging therapeutic agent for human ischemic cardiovascular diseases, clinical conditions and requirements must be fulfilled before any clinical trials are initiated [249]. They include the safety of hiPSC-derived ECs as a drug, and risk assessments [250]. Prior to using hiPSC-ECs in human investigational applications, stringent preclinical analyses are required, following the government’s Chemistry, Manufacturing and Controls (CMC) recommendations for cell-based therapy. Importantly, absence of residual undifferentiated iPSCs from hiPSC-derived ECs must be ensured, because teratoma formation from residual undifferentiated iPSCs has been one of the major concerns in stem cell-based therapy [251]. Side-by-side experiments by Gutierrez-Aranda et al. showed that tumorigenesis with hiPSCs occurs even faster and more aggressively than with hESCs, regardless of the site of injection [252]. In association with the safety concerns, the identity and purity of hiPSC-EC products must also be assessed. HLA typing [253] and biodistribution of implanted cells [254] should be addressed. In addition, reproducibility of the biologics should be established without considerable batch-to-batch variation in large-scale cell production. Moreover, in vitro and in vivo functionality tests should be conducted to ensure the quality control of hiPSC-derived ECs. Cell viability is important. Lastly, it is vital to optimize the procedure to obtain a sufficient number of functional hiPSC-derived ECs, considering industrial-scale production, in the most efficient and cost-effective ways.

Over the years, poor cell survival and engraftment have been hurdles to overcome in many cell types; however, recent studies have shown improved survival of hiPSC-derived ECs with or without biomaterials. Many studies have used various biomaterials to encapsulate hiPSC-derived ECs and have shown improved cell survival [73,236,237,255]. Interestingly, Lee et al. [73] demonstrated the relatively longer (approximately ten months) survival of injected hiPSC-derived ECs in ischemic hindlimbs even without biomaterials, and showed improved survival and neovascularization when hiPSC-ECs were encapsulated in a nanomatrix gel. While it is clear that biomaterials enhance cell viability and survival in the ischemic region and sustain therapeutic performance of transplanted hiPSC-derived vascular cells, it is imperative to evaluate the clinical compatibility and safety of biomaterials for cell transplantation.

Conversely, the use of hiPSC-derived SMCs to enhance recovery from ischemia and promote tissue regeneration has recently been under investigation [239,240]. More studies are needed to ensure their therapeutic effects. Therefore, we address the following considerations for hiPSC-derived SMCs. There is, of course, a major question regarding the ideal SMC phenotype for stimulating neovascularization to re-build a functional blood vessel. In a series of early studies between 1999 and 2006, many evaluated the effects of transplanted SMCs on animal models of cardiovascular diseases. Five studies by Li et al. [256], Sakai et al. [257], Fujii et al. [258], Liu et al. [259], and Nakamura et al. [260] all showed that transplanted rat SMCs prevented cardiac dilation and improved heart function after a myocardial infarction. Meanwhile, Yoo et al. [261] and Yoo et al. [262] isolated SMCs from the ductus deferens of hamsters and determined that SMC transplantation improved left ventricular function and limited ventricular dilatation in a hamster model of dilated cardiomyopathy. As exemplified in these animal studies, in vitro characterization of cultured SMCs was assessed by staining for either ACTA2 or MYH11. Therefore, the findings provide an important clue that a contractile SMC phenotype might be more suitable, but the ideal SMC phenotype for clinical use must be addressed before hiPSC-derived SMCs become a clinical reality. Secondly, as previously mentioned, SMCs undergo remarkable phenotypic switching that allows rapid adaptation to external cues [224]. While maintaining either a synthetic or contractile SMC phenotype in cultured hiPSC-derived SMCs is relatively manageable, little is known about the fate of transplanted hiPSC-derived SMCs in vivo. Ultimately, long-term follow-up studies in animals should be conducted to determine the behavior and safety of transplanted hiPSC-derived SMCs.

## Figures and Tables

**Table 1:** Criteria to define the EC identity.

Characteristics	Methods	Expected Features
Cell morphology	Bright-field/phase-contrast microscopy	Cobblestone-like shape with a single nucleus
Increased expression of EC-specific markers	RT-PCR Western blot Flow cytometry Immunocytochemistry	KDR, CDH5, VWF, PECAM1, TEK, and NOS3
In silico analysis	Microarray RNA-sequencing Single-cell RNA-sequencing Single-cell ATAC-sequencing	Upregulation of EC-specific genes Enriched GO terms related to ECs Comparison with human primary ECs
In vitro functionality	Immunocytochemistry	NO production Uptake of acetylated LDL
	RT-PCR Western blot ELISA	Elevated expression of angiogenic factors, including VEGFA, ANGPT1, IGF1, and FGF2
	Cell migration	Increased overall motility
	Tube formation	Tube-like structure formation

**Table 2:** Criteria to define contractile SMC identity.

Characteristics	Methods	Expected Features
Cell morphology	Bright-field/phase-contrast microscopy Transmission electron microscopy	Spindle-shaped with a single nucleus Enhanced filamentous patterns and dense bodies
Increased expression of contractile SMC-specific markers	RT-PCR Western blot Flow cytometry Immunocytochemistry	ACTA2, TAGLN, CALD1, DES, CNN1, MYH11, and SMTN
	Reporter transgene	Contractile SMC-specific gene promoter
(Optional) Detection of contractile SMC fate determining transcription factors	RT-PCR Western blot Flow cytometry Immunocytochemistry	SRF, MYOCD, MRTFA and B, CRIP1 and 2, and PITX2
Transcriptome analysis	Microarray RNA-sequencing Single-cell RNA-sequencing	Upregulation of contractile SMC-specific genes Enriched GO terms related to contractile SMCs Comparison with primary human SMCs
In vitro functionality	Contraction Intracellular calcium release Force generation	Responding to vasoactive agents

**Table 3:** Summary on therapeutic applications of hiPSC-derived vascular cells.

Cell Type	Number of Cells/Head	Delivery Method	Species (Sex)	Animal Model	Reference
hiPSC-derived EC	5 × 10^5^	Intramuscular injection	NOD/SCID mouse (male)	Hindlimb ischemia	[70]
	1 × 10^6^	Intramuscular injection	NOD/SCID mouse (male)	Hindlimb ischemia	[234]
	3 × 10^6^	Intramuscular injection	SCID mouse (male)	Hindlimb ischemia	[235]
	5 × 10^5^	Intramuscular injection	NOD/SCID mouse (male)	Hindlimb ischemia	[236]
	1 × 10^6^	Intramuscular injection	NOD/SCID mouse (male)	Hindlimb ischemia	[237]
	2 × 10^5^	Intramuscular injection	Athymic nude *Foxn1^nu^* mouse (male)	Hindlimb ischemia	[73]
	1 × 10^6^	Intramyocardial injection	NOD/SCID mouse (female)	Myocardial infarction	[238] *
hiPSC-derived pericyte	2 × 10^6^	Intramuscular injection	CD-1 nude mouse (male)	Hindlimb ischemia	[239]
hiPSC-derived SMC	1 × 10^6^	Intramuscular injection	Athymic nude *Foxn1^nu^* mouse (male)	Hindlimb ischemia	[240]

* Differentiated ECs were derived from porcine iPSCs. NOD/SCID: nonobese diabetic/severe combined immunodeficiency; SCID: severe combined immunodeficiency; CD-1: immunodeficient developed from the transfer of the nude gene from Crl:NU-*Foxn1nu* at Charles River Laboratories, Wilmington, MA, USA.

## Data Availability

Not applicable.

## Abbreviations

Official NCBI gene symbols are used in this review for the sake of consistency.

AcLDL	Acetylated low-density lipoprotein
ACTA2	Actin alpha 2, smooth muscle
ANGPT2	Angiopoietin-2
APLNR	Apelin receptor
ATAC-seq	Assay for transposase-accessible chromatin using sequencing
B3GAT1	Beta-1,3-glucuronyltransferase 1
BLI	Bioluminescence imaging
BMP4	Bone morphogenetic protein 4
CALD1	Caldesmon 1
CB-ECFC	Cord-blood endothelial colony-forming cell
CD34	CD34 molecule
CD44	CD44 molecule (Indian blood group)
CDH5	Cadherin 5
CMC	Chemistry, Manufacturing, and Control
cAMP	Cyclic adenosine monophosphate
CNN1	Calponin 1
COL1A1	Collagen type I alpha 1 chain
NR2F2	Nuclear receptor subfamily 2 group F member 2
CRIP1 and 2	Cysteine rich protein 1 and 2
DES	Desmin
DLL4	Delta like canonical Notch ligand 4
EB	Embryoid body
EC	Endothelial cell
EFNB2	Ephrin B2
EGF	Epidermal growth factor
eGFP	Enhanced green fluorescent protein
ELISA	Enzyme-linked immunosorbent assay
ENG	Endoglin
EOMES	Eomesodermin
EPHB4	EphB4
ERG	ETS-related gene
FACS	Fluorescence-activated cell sorting
FBS	Fetal bovine serum
FGF	Fibroblast growth factor
FGF2	Fibroblast growth factor 2
FOXF1	Forkhead box F1
GBX2	Gastrulation brain homeobox 2
GJA1	Gap junction protein alpha 1
GMP	Good manufacturing practice
GO term	Gene Ontology term
GSK3β	Glycogen synthase kinase 3 beta
hBM	Human bone marrow mononuclear cell
hESC	Human embryonic stem cell
hiPSC	Human induced pluripotent stem cell
HUVEC	Human umbilical cord endothelial cell
iEnd	induced EC via transfection of fibroblasts with POU5F1 and KLF4; ISL1, ISL LIM homeobox 1
ITGA2	Integrin subunit alpha 2
KDR	Kinase insert domain receptor
KEGG pathway	Database of metabolic pathways from Kyoto Encyclopedia of Genes and Genomes
KLF4	Kruppel like factor 4
LIN28A	Lin-28 homolog A
LMOD1	Leiomodin 1
Lrp5 and 6	Low density lipoprotein receptor-related protein 5 and 6
MAP2K7	Mitogen-activated protein kinase 7
MCAM	Melanoma cell adhesion molecule
MEOX1	Mesenchyme homeobox 1
MITCH-PEG	Mixing-Induced Two-Component Hydrogels combined with PolyEthylene Glycol hybrid hydrogel
MIXL1	Mix paired-like homeobox 1
MRTFA and B	Myocardin related transcription factor A and B
MYC	MYC proto-oncogene, bHLH transcription factor
MYH11	Myosin heavy chain 11
MYH11b	Myosin, heavy chain 11b, smooth muscle (Danio rerio (zebrafish))
MYLK	Myosin light chain kinase
MYOCD	Myocardin
NANOG	Nanog homeobox
NES	Nestin
NGFR	Nerve growth factor receptor
NKX2-5	NK2 homeobox 5
NO	Nitric oxide
NOTCH1	Notch receptor 1
NOS3	Nitric oxide synthase 3
NRP1	Neuropilin 1
NT5E	5′-Nucleotidase ecto
OLIG3	Oligodendrocyte transcription factor 3
PAX1, 3, and 7	Paired box 1, 3, and 7
PDGFB	Platelet-derived growth factor subunit B
PDGFR⍺ and β	Platelet-derived growth factor receptor alpha and beta
PECAM1	Platelet and endothelial cell adhesion molecule 1
PI3K	Phosphatidylinositol-4,5-biphosphate 3-kinase
PITX2	Paired like homeodomain 2
POU5F1	POU class 5 homeobox 1
RA	Retinoic acid
ROCK	Rho-associated protein kinase
RT-PCR	Reverse transcription polymerase chain reaction
SHIELD	Shear-thinning Hydrogel for Injectable Encapsulation and Long-term Delivery
SMC	Smooth muscle cell
SMTN	Smoothelin
SOX1, 2, 9, and 10	SRY-box transcription factor 1, 2, 9, and 10
SRF	Serum response factor
SYNPO2	Synaptopodin 2
TAGLN	Transgelin
TBX6 and T	T-box transcription factor 6 and T
TCF15	Transcription factor 15
TEK	Tyrosine kinase
TFAP2A	Transcription factor AP-2 alpha
TGF-β	Transforming growth factor-beta
THY1	Thy-1 cell surface antigen
VEGF	Vascular endothelial growth factor
VEGFA	Vascular endothelial growth factor A
VIM	Vimentin
VWF	von Willebrand factor
Wnt	Wingless/Integrated
Wnt3	Wingless-type MMTV integration site family, member 3
